# Recent advances in amorphous electrocatalysts for oxygen evolution reaction

**DOI:** 10.3389/fchem.2022.1030803

**Published:** 2022-09-27

**Authors:** Jinkyu Park, Seonggyu Lee, Seongseop Kim

**Affiliations:** ^1^ Department of Chemical and Biomolecular Engineering, Korea Advanced Institute of Science and Technology (KAIST), Daejeon, South Korea; ^2^ Department of Chemical Engineering, Kumoh National Institute of Technology, Gumi, South Korea; ^3^ School of Chemical Engineering, Clean Energy Research Center, Jeonbuk National University, Jeonju, South Korea

**Keywords:** amorphous electrocatalysts, oxygen evolution reaction, water splitting, electrolysis, amorphous material

## Abstract

Oxygen evolution reaction (OER) has attracted great attention as an important half-reaction in the electrochemical splitting of water for green hydrogen production. However, the inadequacy of highly efficient and stable electrocatalysts has impeded the development of this technology. Amorphous materials with long-range disordered structures have exhibited superior electrocatalytic performance compared to their crystalline counterparts due to more active sites and higher structural flexibility. This review summarizes the preparation methods of amorphous materials involving oxides, hydroxide, phosphides, sulfides, and their composites, and introduces the recent progress of amorphous OER electrocatalysts in acidic and alkaline media. Finally, the existing challenges and future perspectives for amorphous electrocatalysts for OER are discussed. Therefore, we believe that this review will guide designing amorphous OER electrocatalysts with high performance for future energy applications.

## Introduction

Current fossil fuel-based energy production has caused serious problems such as climate change and environmental pollution. To solve these problems, the demand for clean and renewable energy sources have been increased. Among the various energy sources, hydrogen is one of the most promising candidates that can substitute fossil fuels because of its abundance, high energy density and zero-emission combustion properties (Park et al., 2019). Most of the hydrogen is mainly produced by a steam reforming of natural gas, which emits a lot of greenhouse gases (Lim et al., 2022), therefore, it should be accompanied by the development of clean hydrogen production systems.

Water electrolysis has been garnering attention as the clean hydrogen production system that converts surplus electrical energy into chemical energy by splitting the water into hydrogen and oxygen without any pollutants. The water electrolysis consists of two coupled half-reactions, which are the hydrogen evolution reaction (HER) in the cathode and the oxygen evolution reaction (OER) in the anode. Between the reactions, OER requires a higher overpotential than HER due to the sluggish kinetics of the complicated reaction mechanism, and significantly lowers the efficiency of the overall water electrolysis system (Guo et al., 2022). Therefore, the development of highly active OER electrocatalysts is one of the most important steps to improve the efficiency of the water electrolysis system and replace fossil fuels with hydrogen.

Over the past few years, substantial efforts have been devoted to enhancing OER activities of the electrocatalysts, for example, the introduction of hetero atoms into the lattice or on the surface to enhance the intrinsic kinetic activity (Ramesh et al., 2018; Youk et al., 2019), and structural engineering of the electrocatalysts to increase the surface area and/or mass transport property (Chen et al., 2018). Among them, the development of amorphous-structured OER electrocatalysts is considered a promising strategy for enhancing the activity; it is based on that the surface of crystalline materials is electrocatalytically activated while becoming amorphous during OER (Tang et al., 2022).

**FIGURE 1:**
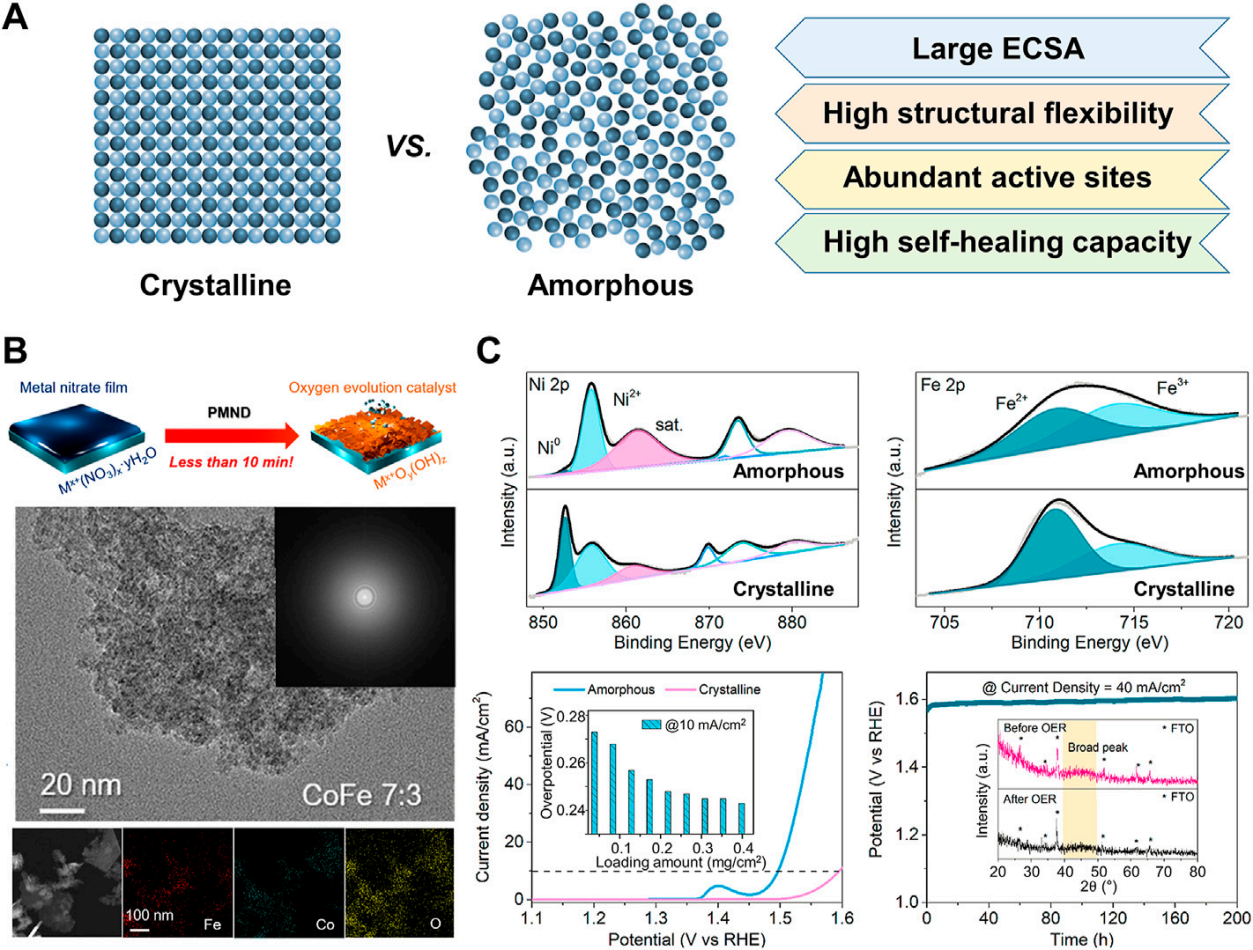
**(A)** Advantages of amorphous electrocatalysts compared to crystalline counterparts in OER **(B)** Precipitating metal nitrate deposition synthesis of amorphous metal oxyhydroxides and TEM of CoFe oxyhydroxide, reproduced with permission from Kim et al. (2019) **(C)** Surface valence states and electrocatalytic performance of amorphous NiFe alloy, reproduced with permission from Cai et al. (2020).

Amorphous materials with long-range disordered structures have exhibited superior electrocatalytic OER performance compared to their crystalline counterparts due to the following advantages (Figure 1A). First, the amorphous structure has a large number of active sites from rich dangling bonds and coordinatively unsaturated sites (Ying and Wang, 2021). Next, the atomic composition can be controlled over a wide range, which makes it possible to find the optimal composition for the best activity (Lemoine et al., 2021). In addition, abundant defects in amorphous materials can improve the diffusion property that facilitates the transportation of reactants and products (Zhai et al., 2021). Moreover, amorphous materials have higher structural flexibility through facile morphology engineering (Zhang D et al., 2021).

In this review, we summarize the various preparation methods of amorphous materials involving oxides, hydroxide, phosphides, sulfides, and their composites, and introduce the latest developments of amorphous OER electrocatalysts in acidic and alkaline media. Finally, the existing challenges facing the development of amorphous OER electrocatalysts and future perspectives are discussed. This review will provide systematic insights and in-depth understanding, and therefore guide the design strategy of amorphous OER electrocatalysts with high performance for future energy applications.

## Amorphous electrocatalysts in alkaline conditions

In alkaline electrolytes, earth-abundant metal catalysts (i.e., non-noble metal) have captured great attention in OER electrocatalysts due to their low cost, excellent cyclability, and tunable structures (Xu et al., 2019). Especially amorphous transition metal oxides have exhibited high OER activities, thus they have been considered promising electrocatalysts in alkaline media (Smith et al., 2013a; Indra et al., 2014). For example, photochemical metal-organic decomposition (PMOD) has been used to prepare amorphous Fe-based metal oxide electrocatalysts on a certain substrate (Smith et al., 2013b). Fe metal-organic precursor films are prepared on fluorine-doped tin oxide (FTO) substrates and are directly photolyzed into amorphous Fe_2_O_3_ by irradiation of 185- and 254 nm. PMOD also can prepare various types of amorphous metal oxides with different compositions. Amorphous mixed-metal oxides of Fe, Co., and Ni (a-FeCoNiO_x_) achieve an overpotential of 230 mV and Tafel slop of 31 mV dec^−1^ in 0.1 M KOH.

The addition of hetero atoms breaks the crystallization of perovskite oxides (ABO_3_) to amorphous phases, generating rich dangling bonds and defects for enhanced OER performance (Yang et al., 2018; Guo et al., 2019). Shao and co-workers introduced FeCl_3_ for both top-down and bottom-up amorphization of LaNiO_3_ by the loss of La ion (Chen G. et al., 2019). For the top-down synthesis of amorphous LaNiFe hydroxide, crystalline LaNiO_3_ was firstly prepared by sol-gel reaction of La and Ni nitrates with EDTA-citrate complexing agent in an aqueous solution. The evaporation at 90°C yielded a resultant gel followed by heat treatment at 800°C to form bulk perovskite powder. The powder was grounded and added in solution with FeCl_3_ with ultrasonication, resulting in amorphous LaNiFe hydroxide. For bottom-up synthesis, La, Fe, and Ni nitrates were dissolved in water and precipitated by excess KOH solution, preparing amorphous LaNiFe hydroxide. Fe octahedra interstitials transform corner-shared octahedra structures of crystallites into edge-shared octahedra with high-valence Ni^3+^ sites. Furthermore, the X-ray absorption spectrum (XAS) reveals the valence state of +3 in the top-down sample, but of +2 in the bottom-up sample. Enhanced OER activity is attributed to the disordered structures that can bond OH^−^ with bridging and terminal geometries. Especially, amorphous NiFeO_x_ prepared by the top-down approach shows an extremely low overpotential of 189 mV at 10 mA cm^−2^, which is lower than that of the crystalline counterpart of 338 mV at 10 mA cm^−2^.

Yu and co-workers exploited a co-precipitation method for scalable synthesis of amorphous metal oxides (Duan et al., 2019b). The supersaturated solution containing Ni, Fe, and Mo precursors precipitated into NiFeMo oxides 515 g of catalysts in one batch within minutes. They also found that the surfaces of the amorphous metal oxides are quickly self-reconstructed during the OER process into metal oxyhydroxides, but the crystalline counterpart shows sluggish reconstruction. Amorphous NiFeMo oxyhydroxide layers have rich oxygen vacancies, enhancing OER activity with 280 mV overpotential at 10 mA cm^−2^ in 0.1 M KOH. Precipitating metal nitrate precursors also can directly transform to amorphous Fe, Co., and Ni-based metal oxyhydroxide on the various substrates (Thangavel et al., 2021). The metal nitrate solutions dropped on the substrates such as FTO, Ni foam (NF), and carbon felt (CF) are precipitated to metal hydroxides in KOH solution and the sequential electrooxidation process synthesizes metal oxyhydroxides (Figure 1B). This method allows the easy control of metal compositions (Kim et al., 2019). Among prepared catalysts with various compositions, the NiFe (2:8) electrocatalyst on NF shows the best OER performance with a low overpotential of 280 mV at 100 mA cm^−2^ in 1.0 M KOH.

To understand the mechanism of the reconstruction of oxides, Xu and co-workers investigated the correlation of Ni substitution and reconstruction of metastable multi-metal oxides to amorphous oxyhydroxide surface via density functional theory (DFT) calculation and experimental characterizations (Duan et al., 2019a). They prepared amorphous ZnCo_2-*x*_Ni_*x*_O_4_ by adding Ni precursor to the solution containing Zn and Co. precursors for crystalline ZnCo_2_O_4_, which was dried at 170°C and calcined at 300°C. The replacement of Ni with Co. in ZnCo_2_O_4_ distorts the crystalline structure, resulting in metastable spinel oxides. The component ratio is tailored in ZnCo_2-*x*_Ni_*x*_O_4_ with different *x*, which shifts the relative position of O p-band versus the metal d-band. The increase of *x* values (*x* > 0.4) renders a higher O p-band center than the metal d-band, leading to active lattice oxygen that is reconstructible into oxyhydroxide on the surface after OER cycling. The ZnCo_1.2_Ni_0.8_O_4_ catalyst exhibits decreased overpotential from 391 to 313 mV at 25 μA cm^−2^ after amorphization, which is comparable to IrO_2_.

Highly active metal oxyhydroxides also can be converted from unstable metal sulfides or phosphides during the anodic oxygen evolution (Cai et al., 2017; Fan et al., 2018; Che et al., 2019; Huang et al., 2020). For example, Wang and co-workers prepared amorphous Ni-based bimetallic sulfide nanosheets with porosity by sulfidation of metal organic frameworks (Qin et al., 2019). First, NiCo-based metal organic framework (MOF) was synthesized using hydrothermal method of the solution containing Ni and Co. nitrates and 2-methyl imidazole at 140°C with same molar ratio of Ni and Co. Then, NiCo-MOF was sulfidized to amorphous holey NiCoS nanosheets in the ethanol solution with thioacetamide solution at 90°C, which can easily control pore size and density via tunable sulfidation time. The NiCoS phase is reconstructed into Ni-Co oxyhydroxide during the OER process. The synergistic effect of 2D morphology, electroactive Ni-Co oxyhydroxides, and unsaturated amorphous structures improve OER activity with an overpotential of 280 mV at 20 mA cm^−2^ in 1.0 M KOH, which is lower than that of crystalline NiCoO nanosheet (370 mV) and IrO_2_ (320 mV). Furthermore, Driess and co-workers reported an amorphous CoP catalyst by a hot injection method (Beltrán-Suito et al., 2019). They prepared two types of CoP materials by using the hot injection and pyrolysis of the molecular precursor. The product from hot injection has amorphous phases, whereas the pyrolyzed product has highly crystalline phases. Both amorphous and crystalline CoP catalysts readily transform to Co. oxyhydroxides on their surface as a result of the dissolution of *p* species. Inner metallic CoP core contributes high electronic conductivity from the active surface to the electrode substrates. More flexible surface and higher active sites of amorphous CoP than that of crystalline promote OER activity as well as HER in the alkaline electrolyte, and also show stable performance over 100 h.

Wu and co-workers employed amorphous iron borate (am-Fe-Bi) nanolattices which are capable of boosting OER (Zhao et al., 2018). Am-Fe-Bi electrocatalysts were self-assembled for the fabrication of nanolattices on NF and cross-linked on the surface via hydrothermal methods. Am-Fe-Bi have suitable values of d-band center and nanolattices expose more active sites, resulting in multi-functional electrocatalysts involving hydrogen and oxygen evolution, and oxygen reduction. The am-Fe-Bi shows superior OER activity with only 158 mV overpotential at 10 mV cm^−2^ that surpass RuO_2_ (228 mV at 10 mV cm^−2^)

Liu and co-workers prepared porous amorphous NiFe alloy with various molar ratios of Ni to Fe for improving OER performances (Cai et al., 2020). They simply synthesized the amorphous NiFe catalysts by reacting Ni and Fe acetates with the NaBH_4_ aqueous solution at room temperature and ambient pressure. The different stoichiometry of Ni and Fe precursors easily controlled the molar ratio of Ni to Fe of the alloy. The amorphous structures expose more active sites by applying anodic potential, which is attributed to their short-range ordering. The degree of surface oxidation of Ni and Fe is higher for the amorphous catalysts than for the crystalline counterpart (Figure 1C). Among the prepared catalysts, amorphous Ni_3_Fe has the highest OER activity with an overpotential of 242 mV at 10 mA cm^−2^ that is and excellent durability in 1.0 M KOH.

Mixed phase boundaries of amorphous and crystalline phases combine the benefits of rich defects, structural flexibility, and enhanced activity of the amorphous phase with stable crystallite in OER (Zhou and Fan, 2020). Song and co-workers investigated heterointerfaces of amorphous-crystalline phase boundary of Co_2_B for OER electrocatalysts (Han et al., 2019). Crystalline Co_2_B catalyst has been prepared by the reaction of CoCl_2_ and NaBH_4_ in 0.1 NaOH solution, and then precipitated powder was pyrolyzed at 600°C under Ar. Adding appropriate amounts of NH_4_F to CoCl_2_ solution during the synthesis procedure of Co_2_B led to fluorin-doped Co_2_B (F-Co_2_B). The fluorination of the catalyst surface is employed for the partial amorphization of Co_2_B, which break crystalline structures by incorporating F. The amorphous-crystalline heterointerfaces decrease energy barriers and increase the charge carrier density, resulting in the promotion of the overall OER kinetics. The engineering of the surface to partial amorphism enhances OER performance of Co_2_B with a superior overpotential of 320 mV at 10 mA cm^−2^ and the Tafel slope of 32 mV dec^−1^ compared with crystalline Co_2_B (370 and 44 mV dec^−1^) and RuO_2_ (350 and 128 mV dec^−1^)

## Amorphous electrocatalysts in acidic conditions

**FIGURE 2:**
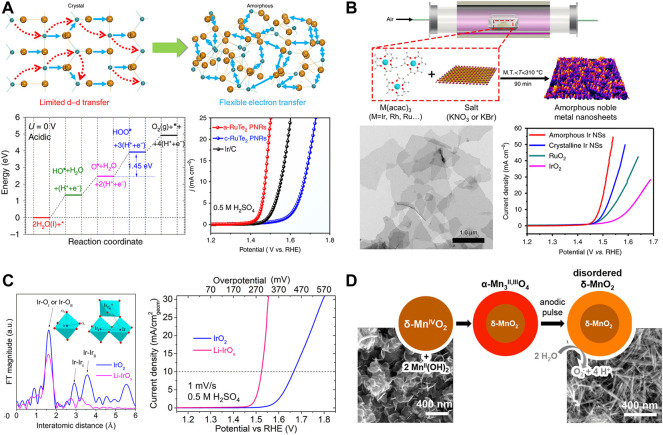
**(A)** Schematic diagram of the electronic activity enhancement, DFT calculation for acidic OER, and electrochemical characterization of a-RuTe_2_ nanorods, reproduced with permission from Wang et al. (2019) **(B)** Synthesis of amorphous Ir nanosheets, and their structural and electrochemical characterization, reproduced with permission from Wu et al. (2019) **(C)** Atomic coordination measurement and electrocatalytic performance of rutile IrO_2_ and amorphous Li-IrO_*x*_, reproduced with permission from Gao et al. (2019) **(D)** Structural transition during amorphization of *δ*-MnO_2_, reproduced with permission from Huynh et al. (2014).

A variety of transition metal-based catalysts has been developed for OER in alkaline electrolytes, whereas catalyst materials in acid electrolytes have been limited due to their instability in acid. Therefore, noble metal catalysts have been extensively explored to be studied in acid conditions. Amorphous Ru-based catalysts have attracted remarkable attention for OER in acid (Delmer et al., 2008; Zhang L et al., 2021). Huang and co-workers reported theoretical results by DFT that the locally flexible Te-bonding configuration of amorphous RuTe_2_ lattices induces the distortion-strain sensitizing Te-pπ coupling (Wang et al., 2019). Amorphous RuTe_2_ porous nanorods were prepared by hydrothermal synthesis at 180°C, in which Cl_3_H_18_N_6_Ru and K_2_TeO_3_ were used as precursors. The distorted Ru-Te bonding enhances electron transfer of Ru sites as well as OER performances (Figure 2A). Amorphous RuTe_2_ porous nanorods exhibited high OER activity with an overpotential of 245 mV in 0.5 M H_2_SO_4_ which is higher than that of crystalline ReTe_2_ (442 mV)and Ir/C (323 mV). Amorphous catalyst also achieved a low voltage of 1.52 V at 10 mA cm^−2^ in water splitting as both anode and cathode in the identical acid solution. Reconstructed amorphization of RuO_2_ promotes OER activity and stability in the harsh acidic condition (Zhuang et al., 2019). Zhang and co-workers prepared amorphous RuO_*x*_ shells on the surface of Ru or Ru-based alloys via electrooxidation process in acid (An et al., 2022). The strong bond strength of Ru leads to the reconstruction of stable amorphous RuO_*x*_ and inhibits steady-state dissolution on the surface. Among various Ru-based alloys (e.g., RuMn, RuCo, RuZn, and RuCr), amorphous RuO_*x*_ on the RuMn surface enhances its durability, which is stable for 720 h at 10 mA cm^−2^ in 0.5 M H_2_SO_4_.

Amorphous Ir-based materials also achieve significantly enhanced anodic performance in OER. For example, Li and co-workers have demonstrated the synthesis of amorphous noble metal nanosheets by the mixtures of metal acetylacetonates (acac) and alkali salts (Figure 2B). They prepared the homogeneous mixture of M (acac)_3_ (M = Ir, Ru, or Rh) and KNO_3_ dissolved in ethanol and water solution and dried for the mixture powder. It was thermal-treated at 300°C under air condition. Removal of KNO_3_ salt by ethanol/water washing yielded noble metal nanosheets. The series of bi-, and trimetallic amorphous nanosheets also have been able to be prepared based on Ir, Rh, and Ru with same procedure, among which amorphous Ir nanosheet exhibited superior OER performance in acid media; its overpotential is 255 mV at 10 mA cm^−2^ which is lower than that of crystalline Ir nanosheet (280 mV), RuO_2_ (301 mV), and IrO_2_ (373 mV) (Wu et al., 2019). Distorted Ir-O bonds in amorphous Ir oxides can affect oxidation states of Ir, and thus boost OER activity (Morimitsu and Oshiumi, 2009; Lee et al., 2018). Photochemically prepared amorphous IrO_*x*_ exhibits higher OER activity than crystalline IrO_*x*_ (Smith et al., 2014). Photoactive precursor, Ir (acac)_3_, was decomposed by light (*λ* < 254 nm) to the amorphous phase at room temperatures, which reaches a Tafel slope of 34 mV dec^−1^ and an overpotential of 220 mV at 10 mA cm^−2^ in 1.0 M H_2_SO_4_.

Hetero atoms have been also employed for the amorphization of crystalline IrO_2_ (Willinger et al., 2017). Liu and co-workers investigated the exploitation of alkali ions, especially lithium ion, to break the long-range order of rigid crystal structure of rutile IrO_2_, resulting in amorphous Li-IrO_*x*_. IrCl_3_ as a precursor reacted with LiOH in an aqueous solution followed by drying at 90°C, and then the resultant powder was calcined at 400°C under air condition, resulting in Li-IrO_*x*_ with the molar ratio of Li to Ir of 0.6:1. Flexible disordered [IrO_6_] octahedrons of Li-IrO_x_ shrink Ir-O bond and promotes outstanding OER activity with higher Ir oxidation states in 0.5 M H_2_SO_4_; an overpotential of Li-IrO_*x*_ (200 mV at 10 mA cm^−2^) is lower than rutile IrO_2_ (300 mV) (Figure 2C) (Gao et al., 2019).

Dissolution of non-noble metal from the noble/non-noble composite oxides can successfully achieve amorphization of oxides (Chen Y. et al., 2019). Cherevko and co-workers prepared highly active amorphous IrO_*x*_ by leaching non-noble elements in Ir-based perovskite materials and compared it with crystalline IrO_2_ and perovskite-based materials (Br_2_PrIrO_6_ and SrIrO_3_). They proposed a stability number (S-number) to evaluate the stability via the matric characterization of the relationship between the evolved oxygen and the dissolved Ir of given catalysts; S-number indicates the amount of evolved oxygen per Ir atom lost in electrolytes. They also suggested the mechanism of the participation of activated oxygen for the formation of oxygen vacancies and boosting the activity of amorphous IrO_x_ (Geiger et al., 2018).

Although non-noble metal catalysts are unstable in acid conditions, some researchers have discovered non-noble metal-based amorphous materials with excellent OER performance. For example, amorphous Mn-based oxides have been kinetically stable in acidic electrolytes due to self-healing properties to compensate for their dissolution (Huynh et al., 2014). Nocera and co-workers reported that sequential cathodization and anodization of *δ*-MnO_2_ generates disordered *δ*-MnO_2_ involving a morphological transition from plates to needles (Figure 2D) (Huynh et al., 2014). Activated amorphous *δ*-MnO_2_ on FTO shows two orders of magnitude higher for OER than untreated *δ*-MnO_2_ in the acidic condition (pH = 2.5) at an overpotential of 600 mV at 0.1 mA cm^−2^, which is much lower than that of unactivated MnO_*x*_ (990 mV). In addition, Xiong and co-workers investigated highly active amorphous metallic NiFeP for OER in both alkaline and acid electrolytes (Hu et al., 2017). The metallic bonds of bulk electrocatalysts enhance macroscopic electrical conductivity, resulting in promoted OER reaction kinetics due to low resistance from the surface to current collectors. Coordinatively unsaturated Ni, Fe, and *p* in amorphous structures contribute to the increase of active sites in which the generated phosphate boosts the intrinsic activity of Fe sites. Amorphous NiFeP achieves an overpotential of 540 mV at 10 mA cm^−2^ and stable performance for over 30 h in the acid electrolyte.

## Conclusion and outlook

This review summarized the recent advances in amorphous materials as OER electrocatalysts. We reviewed the synthesis of various amorphous materials (e.g., oxides, oxyhydroxides, sulfides, phosphides, and alloys) and their OER performances in acid and alkaline conditions. The structural flexibility of amorphous materials provides the possibility to expose more active sites for large electrochemical surface area, and geometrically boosts charge and mass transfer, leading to remarkably improved OER activity compared to crystalline counterparts. The rich intrinsic defect and dangling bonds of amorphous structures also enhance electrocatalytic properties.

Despite the promising achievements of amorphous electrocatalysts, some challenging issues still remain to be resolved. 1) The thermal instability of amorphous phases has limited the methodologies to design efficient catalysts with precise control of morphology (i.e., nanostructure, particle morphology and size, and porous structure) and chemical composition. In this aspect, the systematic research that is possible to successfully fabricate amorphous materials is highly required for designing optimized OER catalysts. 2) The exact active sites in amorphous electrocatalysts remain elusive which means that further studies are to be carried out to understand their reaction and mechanism. Unclarified active sites of amorphous materials are ascribed to *in-situ* transformations of amorphous materials into new amorphous phases during the electrooxidation process. Moreover, unclear atomic arrangements and structural models also make it difficult to elucidate the OER mechanism on amorphous structures through simulations. Hence, for a greater understanding of the active sites of amorphous materials, the cooperation of multiple measurement techniques (e.g., STEM, XAFS, Raman, and XPS) is highly desirable. Furthermore, operando characterizations should be useful to investigate *in-situ* surface amorphization of electrocatalysts in OER operation. Fulfilling rational synthesis methods and a clear understanding of the electrocatalytic properties of amorphous materials can extend their merits beyond OER to electrocatalytic applications such as the oxygen reduction reaction, CO_2_ reduction, and N_2_ reduction.
